# C1q/TNF-Related Protein 9 Promotes Revascularization in Response to Ischemia *via* an eNOS-Dependent Manner

**DOI:** 10.3389/fphar.2020.01313

**Published:** 2020-08-21

**Authors:** Shukuro Yamaguchi, Rei Shibata, Koji Ohashi, Takashi Enomoto, Hayato Ogawa, Naoya Otaka, Mizuho Hiramatsu-Ito, Tomohiro Masutomi, Hiroshi Kawanishi, Toyoaki Murohara, Noriyuki Ouchi

**Affiliations:** ^1^Department of Cardiology, Nagoya University Graduate School of Medicine, Nagoya, Japan; ^2^Department of Advanced Cardiovascular Therapeutics, Nagoya University Graduate School of Medicine, Nagoya, Japan; ^3^Department of Molecular Medicine and Cardiology, Nagoya University Graduate School of Medicine, Nagoya, Japan

**Keywords:** CTRP9, eNOS, angiogenesis, ischemia, endothelial cell

## Abstract

Strategies to promote revascularization are valuable for ischemic cardiovascular disease. Although C1q/TNF-related protein (CTRP) 9 is an adiponectin paralog with protective properties against cardiometabolic disorders, the role of endogenous CTRP9 in endothelial function is largely unknown. This study aimed to investigate the effects of CTRP9 on revascularization processes and dissected the potential mechanisms. CTRP9-knockout (KO) and wild-type (WT) mice were subjected to unilateral hindlimb ischemic surgery. CTRP9-KO mice exhibited impaired blood flow recovery and decreased capillary density in the ischemic limb compared with WT mice. In both CTRP9-KO and WT mice, systemic delivery of an adenoviral vector expressing CTRP9 (Ad-CTRP9) accelerated blood flow recovery. Treatment with recombinant CTRP9 protein increased network formation and migration of cultured human umbilical vein endothelial cells (HUVECs). CTRP9 promoted the phosphorylation of AMP-activated kinase (AMPK), Akt, and endothelial nitric oxide synthase (eNOS) in HUVECs. CTRP9-KO mice also showed reduced phosphorylation levels of AMPK, Akt, and eNOS in the ischemic limbs compared with WT mice. Furthermore, blockade of AMPK or Akt signaling pathway reversed the CTRP9-stimulated eNOS phosphorylation in HUVECs. Treatment with the NOS inhibitor significantly reduced CTRP9-stimulated network formation and migration of HUVECs. Of note, Ad-CTRP9 had no effects on blood flow of the ischemic limb in eNOS-KO mice. These results indicated that CTRP9 promotes endothelial cell function and ischemia-induced revascularization through the eNOS-dependent mechanism, suggesting that CTRP9 represents a target molecule for treatment of ischemic vascular diseases.

## Introduction

There is an increasing number of patients with peripheral arterial disease (PAD) worldwide. Hence, a large number of PAD patients with critical limb ischemia (CLI) require amputation of the affected limbs, which causes not only reduced quality of life but also a decline in life span (Criqui and Aboyans, 2015). Therefore, therapeutic strategies to promote collateral vessel formation and angiogenesis in such patients are important to salvage ischemic tissue.

C1q/TNF-related protein (CTRP) family contains a collagen-like domain and a C1q-like domain and are classified as adiponectin paralogs owing to their structural similarities to adiponectin (Ouchi and Walsh, 2012). In addition, like adiponectin, several CTRP family members, including CTRP1, CTRP9, and CTRP12/adipolin, are expressed mainly in adipose tissues, and function as fat-derived secreted factors (Ouchi and Walsh, 2012; Shibata et al., 2017). Among them, CTRP9 has the highest amino acid sequence homology to adiponectin (Wong et al., 2009).

Recent several reports indicated that CTRP9 exerts beneficial effects on the cardiovascular system. CTRP9 enhances endothelium-dependent vasorelaxation and attenuates high glucose-induced endothelial oxidative damage (Zheng et al., 2011; Cheng et al., 2016). We have demonstrated that administration of CTRP9 attenuates the neointimal hyperplasia in response to vascular injury in wild-type (WT) mice (Uemura et al., 2013). Overexpression of CTRP9 also reduced atherosclerotic lesion formation in apolipoprotein E knockout mice fed a high-fat diet (Huang et al., 2019). We and others have shown that systemic delivery of CTRP9 attenuates myocardial infarct size in WT or diabetic mice after ischemia-reperfusion injury (IRI) (Kambara et al., 2012; Su et al., 2013). Furthermore, we have reported that CTRP9-knockout (KO) mice exhibit increased myocardial infarct size and exacerbated cardiac dysfunction after IRI compared with WT mice (Kambara et al., 2015). However, nothing is known about the role of endogenous CTRP9 in regulation of angiogenesis *in vivo*. This study aimed to investigate the effects of CTRP9 on ischemia-induced revascularization processes *in vivo* using both loss- and gain-of-function genetic manipulations.

## Methods

### Materials

The following primary antibodies were purchased from Cell Signaling Technology (MA, USA): phospho-AMP-activated kinase (AMPK) (Thr172) antibody, AMPK antibody, phospho-Akt (Ser473) antibody, Akt antibody, phospho-endothelial nitric oxide synthase (eNOS) (Ser1177) antibody, eNOS antibody, and α-tubulin antibody. Recombinant CTRP9 was purchased from Aviscera Bioscience (CA, USA). Compound C and *N*_ω_-Nitro-L-arginine methyl ester hydrochloride (L-NAME) were purchased from MilliporeSigma (MA, USA). LY294002 was purchased from FUJIFILM Wako Pure Chemical Corporation (Osaka, Japan). Adenoviral vectors containing the gene for β-galactosidase (Ad-β-gal) and full-length human CTRP9 (Ad-CTRP9) were prepared as previously described (Kambara et al., 2012).

### Mouse Model of Revascularization

WT, CTRP9-knockout (CTRP9-KO), and eNOS-knockout (eNOS-KO) mice in a C57/BL6 background were used in this study (Kondo et al., 2009b; Kambara et al., 2015). Study protocols were approved by the Institutional Animal Care and Use Committee in Nagoya University. Mice aged 8–10 weeks were subjected to unilateral hindlimb surgery under anesthesia with sodium pentobarbital (50 mg/kg intraperitoneally). In this model, the entire left femoral artery and vein were excised surgically. The 7 × 10^8^ infectious units (ifu) of Ad-CTRP9 or Ad-β-gal were injected into the jugular vein at 5 days before surgery.

### Laser Doppler Blood Flow Analysis and Clinical Score

A laser Doppler blood flow (LDBF) analyzer (Moor LDI, Moor Instruments) was used to measure hindlimb blood flow immediately before surgery and on postoperative days 3, 7, 14, and 28. LDBF analysis was performed on the legs and feet. Blood flow was displayed as changes in the laser frequency using different color pixels. After scanning, stored images were analyzed to quantify blood flow. To avoid data variations because of ambient light and temperature, hindlimb blood flow was expressed as the ratio of left (ischemic) to right (nonischemic) LDBF (Kondo et al., 2009b; Maruyama et al., 2012). We also assess clinical score in mice as determined by an index of severity of limb ischemia. 0, normal; 1, pale foot or gait abnormalities; 2, less than half of foot is necrotic; 3, more than half of foot is necrotic without lower leg necrosis; 4, more than half of foot is necrotic with some lower leg necrosis; 5, necrosis or autoamputation of the entire lower limb (Yu et al., 2005).

### Capillary Density Analysis

Capillary density within the thigh adductor muscle was quantified by histological analysis as previously described. Muscle samples were imbedded in OCT compound (Sakura Finetek, Tokyo, Japan) and snap frozen in liquid nitrogen. Tissue slices (7 μm in thickness) were prepared and stained with CD31 (PECAM-1, Becton, Dickinson and Company, NJ, USA) followed by treatment with Alexa Fluor 594-conjugated secondary antibody to detect CD31. The signals were detected and analyzed using a fluorescence microscopy. Ten to 15 randomly chosen microscopic fields from 3 to 4 different sections in each tissue block were examined for the presence of CD31-positive capillary endothelial cells. Capillary density was expressed as the number of CD31-positive cells per muscle fiber (Kondo et al., 2009b; Maruyama et al., 2012).

### Cell Culture

Human umbilical vein endothelial cells (HUVECs) were cultured in endothelial cell growth medium-2 (EGM-2, Lonza, Basel, Switzerland). Before each experiment, cells were placed in endothelial cell basal medium-2 (EBM-2, Lonza, Basel, Switzerland) with 0.5% fetal bovine serum (FBS) for serum starvation for 8–24 h. Experiments were performed by the addition of the indicated amount of recombinant CTRP9 or vehicle for the indicated lengths of time. In some experiments, HUVECs were treated with Compound C (10 µmol/L), LY294002 (10 µmol/L), L-NAME (1 mmol/L), or vehicle (PBS or Dimethyl sulfoxide,DMSO) for 15–60 min.

### Network Formation Assay

The formation of vascular-like structures by HUVECs on growth factor-reduced Matrigel (Corning, NY, USA) was assessed according to the manufacturer’s instructions. HUVECs were seeded in coated plates at 2.0 × 10^4^ cells/cm^2^ in EBM-2 medium and incubated at 37°C for 12–17 h. An inverted phase contrast microscope (Olympus, Tokyo, Japan) was used to assess network formation and photomicrographs were taken at ×40 magnification. The degree of tube formation was quantified by measuring the network area of tubes using the ImageJ software (Ouchi et al., 2004; Maruyama et al., 2012).

### Migration Assay

A modified Boyden chamber assay was used to measure migration activity. Serum-starved cells were trypsinized and resuspended in EBM-2 with 0.5% FBS. Cell suspensions (250 µl, 2.0 × 10^4^ cells/well) were added to the Transwell insert (6.5 mm diameter, 8.0 µm pore size, Corning, NY, USA). Then 750 µl of EBM-2 with 0.5% FBS supplemented with CTRP9 (10 µg/ml) or PBS (control) were added to the lower chamber and incubated for 17 h. Migrated cells on the lower surface of the membrane were fixed, stained with Giemsa stain solution and DAPI solution (FUJIFILM Wako chemicals, Osaka, Japan);, and quantified (Ouchi et al., 2004; Maruyama et al., 2012).

### Western Blot Analysis

Tissue and cell samples were prepared in lysis buffer containing 1 mM PMSF (Millipore Sigma, MA, USA). Pierce BCA protein assay kit (Thermo Fisher Scientific, MA, USA) was used to calculate the protein concentration. Equal amounts of proteins or plasma (2.0 μl) were separated by denaturing SDS-PAGE. Proteins were transferred onto PVDF membranes (Bio-Rad, CA, USA) and probed with the primary antibody followed by incubation with the HRP-conjugated secondary antibody. ECL™ Plus or ECL™ Prime system (GE Healthcare, IL, USA) was used to detect protein signal. The expression level was determined by measurement of the corresponding band intensities by using ImageJ software, and the relative values of the phosphorylated protein were expressed relative to total protein signal. Blood sample was collected by heart puncture from mice that were fasted for 12 h (Maruyama et al., 2012).

### Statistical Analysis

Data are expressed as mean ± SD. ANOVA followed by Tukey’s HSD test and unpaired Student’s t-test was used for statistical analysis. p <.05 was considered statistically significant.

## Results

### CTRP9-KO Mice Show Reduction of Ischemia-Induced Revascularization

**Figure 1:**
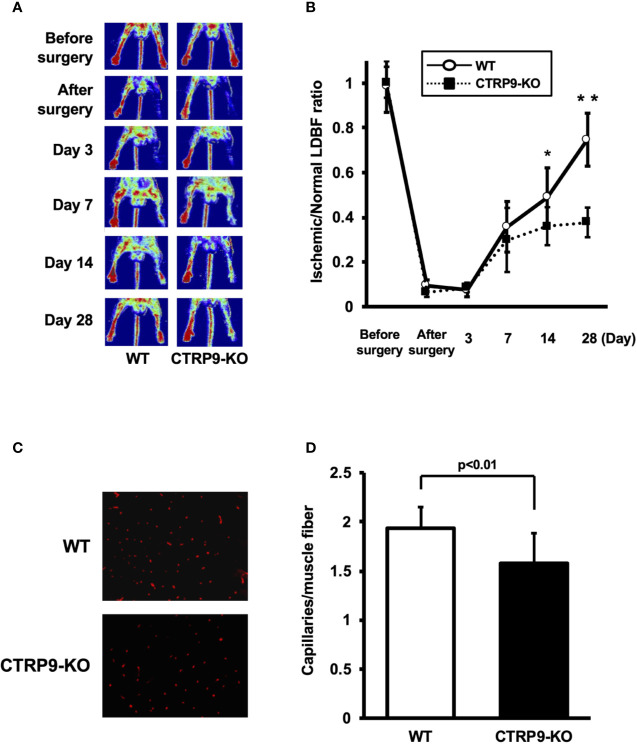
CTRP9-KO mice show reduction of perfusion recovery and capillary density in ischemic limbs. **(A)** Representative laser Doppler blood flow (LDBF) images in ischemic limb of CTRP9-KO or WT mice. A low perfusion signal (dark blue) was observed in the ischemic hindlimb of CTRP9-KO mice, whereas a high perfusion signal (red) was detected in WT mice at postoperative days 14 and 28. **(B)** Quantitative analysis of the ischemic to nonischemic LDBF ratio in the WT and CTRP9-KO mice before surgery and at different time points after surgery. Results are shown as the mean ± SD (n = 7-8 mice for each group). *p < .05, **p < .01 vs CTRP9-KO mice. **(C)** Fluorescence staining of ischemic tissues with anti-CD31 monoclonal antibody (red) at postoperative day 28. **(D)** Quantitative analysis of capillary density in WT and CTRP9-KO mice on postoperative day 28. Capillary density was expressed as the number of capillaries per muscle fiber. Results are presented as mean ± SD (n = 3 mice for each group).

LDBF analysis was performed before surgery and at postoperative days 3, 7, 14, and 28 to evaluate blood flow recovery after ligation of the femoral artery of WT and CTRP9-KO mice. Representative images of blood flow measured by LDBF are shown in **Figure 1A**. In WT mice, hindlimb blood flow perfusion fell precipitously after surgery, remained impaired for 3 days, increased to 36% of the nonischemic limb by day 7, and ultimately returned to 75% of the nonischemic limb by day 28 (**Figure 1B**). In contrast to WT mice, blood flow in the ischemic hindlimb was markedly reduced in CTRP9-KO mice compared with the level in the nonischemic hindlimb at postoperative days 14 and 28 (p <.05 and p <.01, respectively).

To investigate the extent of revascularization at the microcirculatory level, capillary density was measured in ischemic tissues by staining with anti-CD31 antibody (Ab). Representative photomicrographs of histological sections stained with anti-CD31 Ab are shown in **Figure 1C**. Quantitative analysis revealed that the capillary density was significantly reduced in CTRP9-KO mice compared with WT mice at postoperative day 28 (**Figure 1D**).

### Systemic Delivery of CTRP9 Increases Blood Flow in Ischemic Limbs

**Figure 2:**
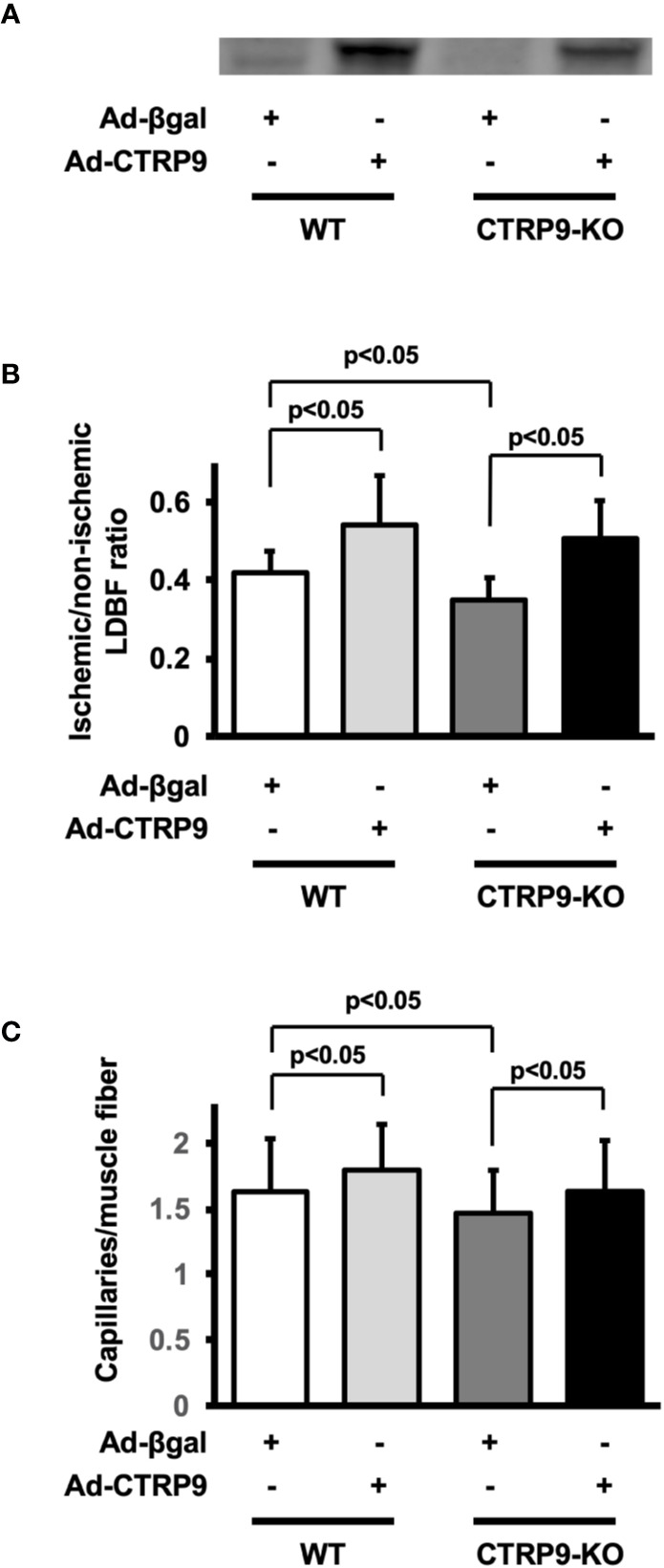
CTRP9 supplementation enhances perfusion of ischemic limbs in WT and CTRP9-KO mice. **(A)** CTRP9 expression in plasma in WT and CTRP9-KO mice after treatment with Ad-CTRP9 or Ad-β-gal (control). Ad-CTRP9 or Ad-β-gal was injected into the jugular vein of WT and CTRP9-KO mice (7 × 10^8^ ifu in each group) at day 5 before surgery. Plasma CTRP9 levels were assessed at day 5 after adenoviral vector injection by Western blot analysis. **(B)** Quantitative analysis of ischemic to nonischemic laser Doppler blood flow (LDBF) ratio in WT and CTRP9-KO mice receiving Ad-β-gal or Ad-CTRP9 on postoperative day 14 (n = 4–9 mice for each group). **(C)** Quantitative analysis of capillary density in WT and CTRP9-KO mice receiving Ad-β-gal or Ad-CTRP9. Capillary density was expressed as the number of capillaries per muscle fiber. Results are presented as mean ± SD (n = 3 mice for each group).

To test whether administration of CTRP9 could modulate revascularization under conditions of ischemia, we systemically injected Ad-CTRP9 or Ad-β-gal as a control through a jugular vein into WT and CTRP9-KO mice at day 5 before surgery. Based on Western blot analysis, administration of Ad-CTRP9 in WT mice significantly increased plasma CTRP9 levels by a factor of 6.6 ± 2.7 at day 5 after adenoviral vector injection compared with Ad-β-gal (**Figure 2A**). Ad-CTRP9-treated CTRP9-KO mice also showed a 6.5 ± 4.6-fold increase in plasma levels of CTRP9 at day 5 after adenoviral vector injection compared with Ad-β-gal-treated WT mice, whereas plasma CTRP9 was not detectable in Ad-β-gal-treated CTRP9-KO mice. Ad-CTRP9-treated WT mice showed a significant increase in limb perfusion at day 14 after hindlimb surgery compared with Ad-β-gal-treated WT mice (**Figure 2B**) (p <.05). Furthermore, systemic delivery of CTRP9 also increased blood flow in CTRP9-KO mice at day 14 after surgery (**Figure 2B**) (p <.05). Ad-CTRP9-treated WT mice had increased capillary density in ischemic limb on postoperative day 14 compared with Ad-β-gal-treated WT mice (**Figure 2C**). Systemic administration of CTRP9 to CTRP9-KO mice also enhanced capillary density at day 14 after surgery (p <.05). Thus, it is likely that CTRP9 administration rescued the impaired revascularization that was seen in CTRP9-KO mice and promoted revascularization in WT mice.

### CTRP9 Promotes Endothelial Network Formation and Migration *In Vitro*

**Figure 3:**
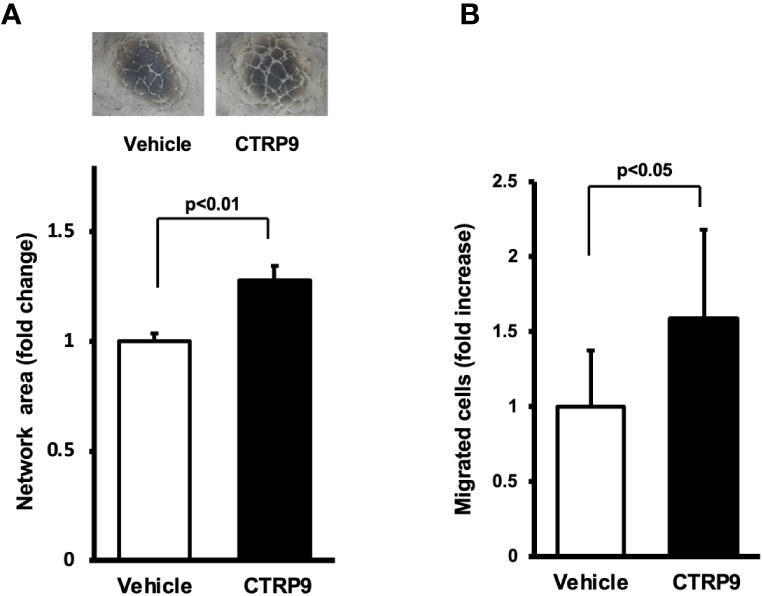
CTRP9 promotes network formation and migration of cultured endothelial cells. **(A)** Endothelial cell network formation after stimulation with CTRP9 or vehicle. After 24 h of serum deprivation, human umbilical vein endothelial cells (HUVECs) were treated with recombinant CTRP9 (10 μg/ml), or PBS (control) followed by culture in Matrigel-coated dishes. Representative photomicrographs of HUVEC network formation is shown (upper panel). Quantitative analyses of the network area of tubes are shown (lower panel) (n = 3 independent culture experiments). **(B)** Endothelial cell migration after stimulation with CTRP9 or vehicle. A modified Boyden chamber assay was performed using HUVECs. HUVECs were treated with CTRP9 (10 µg/ml), or PBS (control). Results are presented as mean ± SD. Results are expressed relative to the values compared with control (n = 8 independent assays).

To examine whether CTRP9 modulates endothelial cell function *in vitro*, HUVECs were plated on a Matrigel matrix followed by treatment with recombinant CTRP9 or vehicle. Treatment with CTRP9 (10μg/ml) significantly increased the network areas of HUVECs (**Figure 3A**). A modified Boyden chamber assay was also performed to test whether CTRP9 affects endothelial migration. CTRP9 significantly stimulated HUVEC migration (**Figure 3B**).

### CTRP9 Stimulates the Phosphorylation of AMPK, Akt, and eNOS

**Figure 4:**
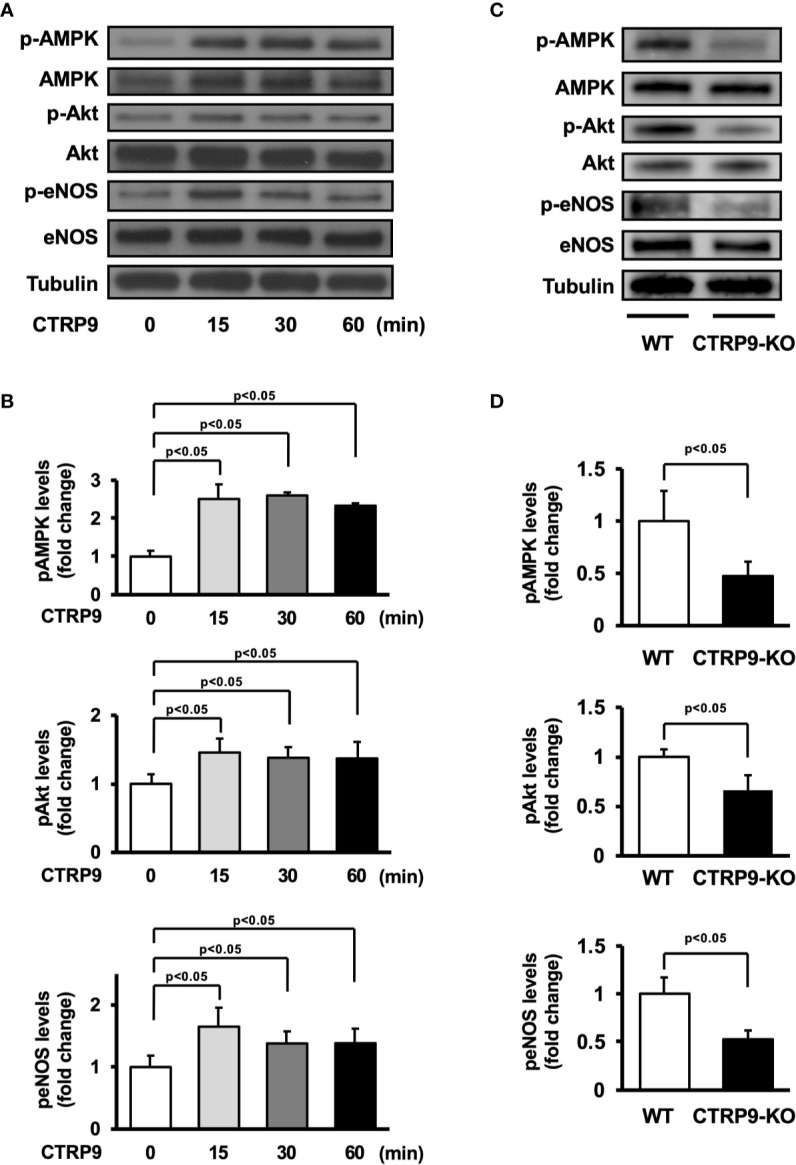
Effect of CTRP9 on the phosphorylation of AMP-activated kinase (AMPK), Akt, and endothelial nitric oxide synthase (eNOS) in endothelial cells and ischemic muscle. **(A)** Time-dependent changes in the phosphorylation of AMPK, Akt, and eNOS signaling in endothelial cells following stimulation with CTRP9. Changes in the phosphorylation of AMPK (p-AMPK), Akt (p-Akt), and eNOS (p-eNOS) after CTRP9 treatment were determined by Western blot analysis. Representative blots are shown. **(B)** Quantitative analysis of relative changes in phosphorylated AMPK, Akt, and eNOS in endothelial cells following stimulation with CTRP9 (n = 3–4 independent culture experiments). **(C)** The phosphorylation of AMPK, Akt, and eNOS in the ischemic muscle of WT and CTRP9-KO mice at day 7 after surgery. Phosphorylation of AMPK, Akt, and eNOS was determined by Western blot analysis. **(D)** Quantitative analysis of relative changes in the phosphoylation of AMPK, Akt, and eNOS in the ischemic muscle of WT and CTRP9-KO mice at day 7 after surgery (n = 3–4 mice for each group). Relative phosphorylation levels were normalized to total protein signal. Relative phosphorylation levels of AMPK, Akt, and eNOS were quantified using ImageJ software. Results are presented as mean ± SD.

AMPK and Akt participate in angiogenic response of endothelial cells (Shiojima and Walsh, 2002; Ouchi et al., 2004; Maruyama et al., 2012). Therefore, we assessed the effects of CTRP9 on the phosphorylation of AMPK and Akt in HUVECs by Western blot analysis (**Figure 4A**). Treatment with recombinant CTRP9 stimulated the phosphorylation of AMPK and Akt in a time-dependent manner (**Figure 4B**). The eNOS phosphorylation level was also assessed because both AMPK and Akt can activate eNOS at Ser-1177 (Fulton et al., 1999; Luo et al., 2000; Ouchi et al., 2004). CTRP9 stimulated the phosphorylation of eNOS (**Figures 4A, B**). CTRP9 had no effects on protein levels of AMPK, Akt, and eNOS.

We next assessed the expression and phosphorylation of AMPK, Akt, and eNOS in ischemic adductor muscle of WT and CTRP9-KO mice by Western blot analysis on postoperative day 7 (**Figure 4C**). Phosphorylation levels of AMPK, Akt, and eNOS in ischemic muscle were significantly lower in CTRP9-KO mice than in WT mice (**Figure 4D**). The expression levels of AMPK, Akt, and eNOS protein in ischemic muscles did not differ between WT and CTRP9-KO mice.

### eNOS Activation Is Essential for CTRP9-Induced Revascularization

**Figure 5:**
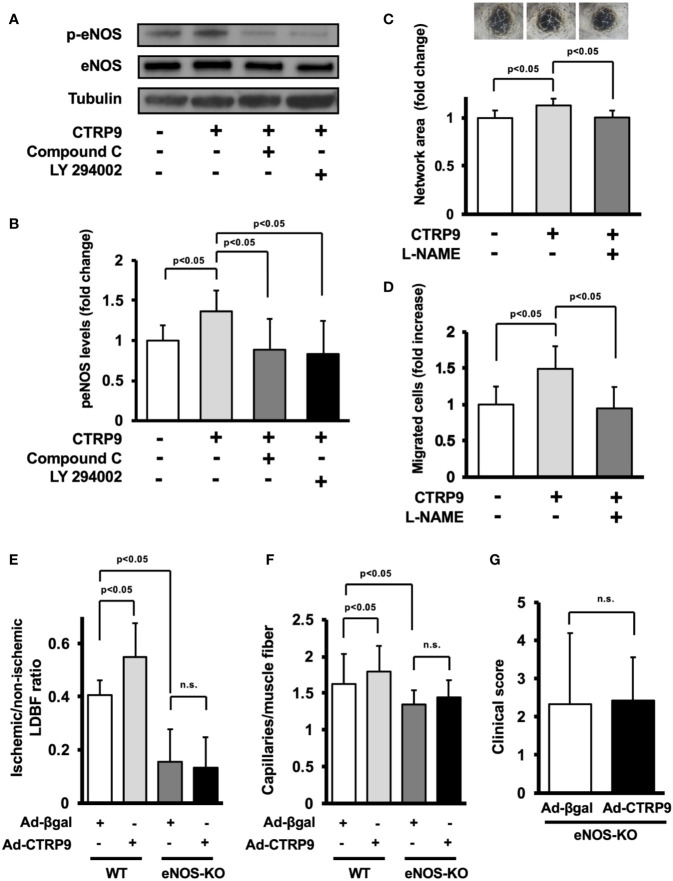
Endothelial nitric oxide synthase (eNOS) signaling is required for angiogenic response to CTRP9. **(A)** Effect of Compound C and LY294002 on CTRP9-stimulated eNOS phosphorylation. Human umbilical vein endothelial cells (HUVECs) were pretreated with Compound C (10 µmol/L), LY294002 (10 µmol/L), or vehicle (DMSO) for 30–60 min and treated with CTRP9 (10 μg/ml) or PBS (control) for 15 min. Phosphorylated eNOS (p-eNOS) was determined by Western blot analysis. **(B)** Quantitative analysis of relative changes in p-eNOS is shown. Relative phosphorylation levels of eNOS were quantified by using ImageJ software. Relative phosphorylation levels were normalized to total protein signal. Results are presented as mean ± SD (n = 5 independent culture experiments). **(C)** Contribution of eNOS to CTRP9-induced network formation of HUVECs. HUVECs were pretreated with L-NAME (1 mmol/L) or vehicle (PBS) and treated with CTRP9 (10 μg/ml), or PBS (control) for 17 h. Representative photomicrographs of HUVEC network formation are shown (upper panel). Quantitative analyses of the network area of tubes are shown. Results are presented as mean ± SD (lower panel) (n = 5–9 independent culture experiments). **(D)** Contribution of eNOS to CTRP9-induced migration of HUVECs. A modified Boyden chamber assay was performed using HUVECs. HUVECs were pretreated with L-NAME (1 mmol/L) or vehicle (PBS) and treated with CTRP9 (10 μg/ml), or PBS (control). Results are presented as mean ± SD (n = 4-5 independent assays). **(E)** Quantitative analysis of ischemic to nonischemic laser Doppler blood flow (LDBF) ratio in Ad-CTRP9 or Ad-β-gal-treated WT and eNOS-KO mice on postoperative day 14 (n = 5–7 mice for each group). **(F)** Quantitative analysis of capillary density in Ad-CTRP9 or Ad-β-gal-treated WT and eNOS-KO mice on postoperative day 14. Capillary density was expressed as the number of capillaries per muscle fiber. Results are presented as mean ± SD (n = 3 mice for each group). **(G)** Clinical score in eNOS-KO mice receiving Ad-CTRP9 or Ad-β-gal as determined by an index of severity of limb ischemia (n = 6-7 mice for each group). ns, not significant.

The potential involvement of AMPK or Akt in CTRP9-induced eNOS phosphorylation *in vitro* was examined by treating HUVECs with AMPK inhibitor Compound C or PI3-kinase inhibitor LY294002 (**Figure 5A**). CTRP9-stimulated phosphorylation of eNOS was reversed by treatment with Compound C or LY294002 (**Figure 5B**). CTRP9-stimulated network formation of HUVECs was diminished by treatment with the NOS inhibitor L-NAME (**Figure 5C**). CTRP9-stimulated migration of HUVECs was also diminished by treatment with L-NAME (**Figure 5D**).

Finally, to examine whether eNOS signaling is required for the actions of CTRP9 on revascularization *in vivo*, we systemically injected Ad-CTRP9 or control Ad-β-gal into eNOS-KO mice 5 days before surgery. eNOS-KO mice showed reduced blood flow on postoperative day 14 after treatment with Ad-β-gal compared with WT mice. In contrast to WT mice, systemic delivery of Ad-CTRP9 did not affect the blood flow in ischemic limbs of eNOS-KO mice on postoperative day 14 (**Figure 5E**). Furthermore, eNOS-KO mice showed reduced capillary density after treatment with Ad-β-gal compared with WT mice. Systemic delivery of Ad-CTRP9 had no effects on the capillary density in ischemic limbs of eNOS-KO mice (**Figure 5F**). We also assessed lower limb function and tissue salvage after surgery using a clinical scoring system. Similarly, the index of severity of tissue ischemia after hindlimb surgery did not differ between Ad-β-gal-treated eNOS-KO and Ad-CTRP9-treated eNOS-KO mice (**Figure 5G**). These data indicated that eNOS activation could be essential for CTRP9-induced revascularization.

## Discussion

**Figure 6:**
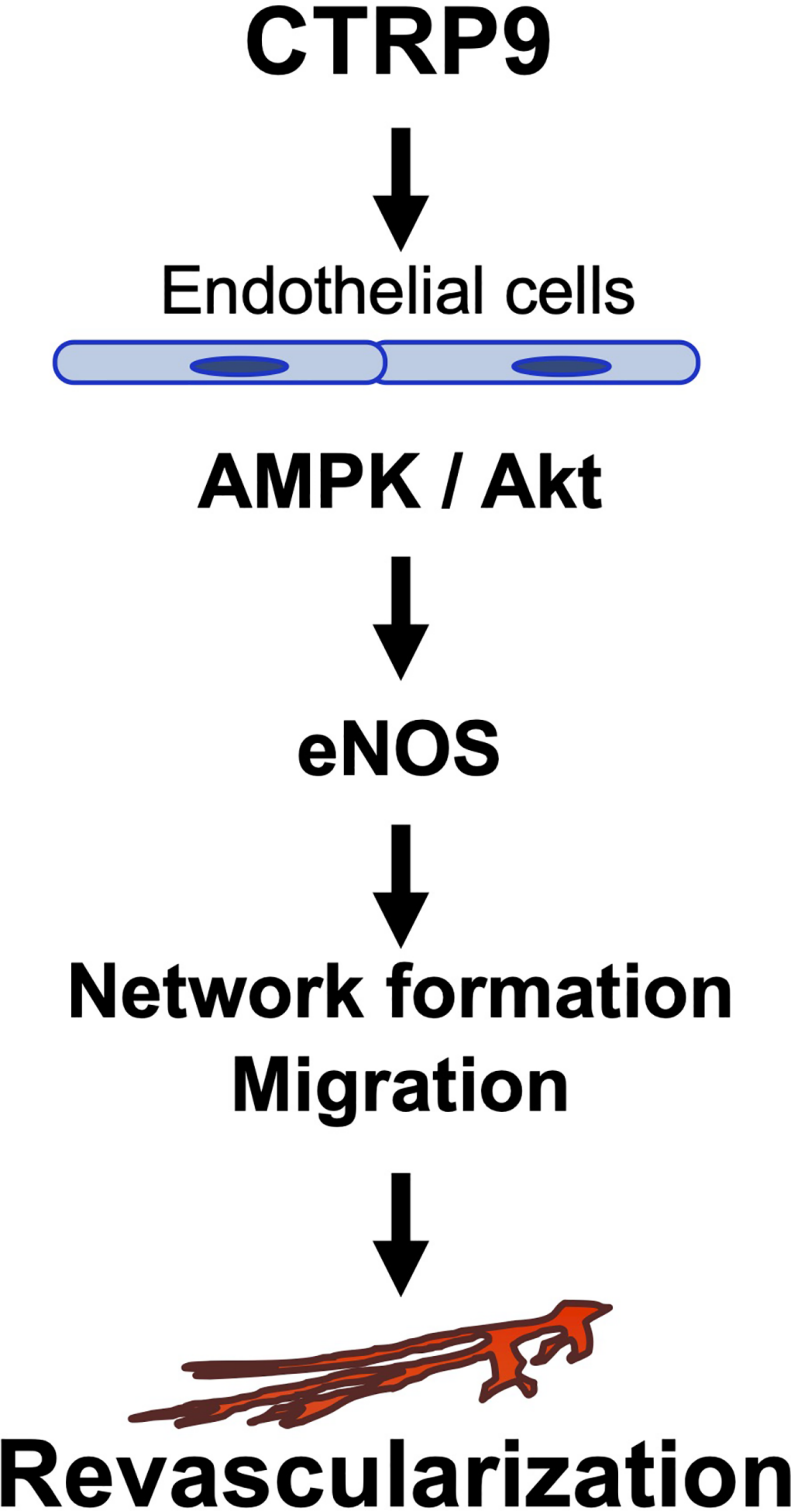
Proposed scheme for the angiogenic actions of CTRP9.

The major findings in this study are as follows: (1) CTRP9 deficiency contributed to impaired revascularization in response to tissue ischemia. (2) Systemic delivery of CTRP9 accelerated blood flow recovery in ischemic muscle. (3) CTRP9 promoted endothelial cell network formation and migration *in vitro*. (4) CTRP9 promoted eNOS activation in endothelial cells through the AMPK or Akt signalling pathway (5) eNOS was involved in the pro-angiogenic effects of CTRP9 *in vitro* and *in vivo*. These data indicated that CTRP9 can stimulate blood vessel growth in ischemic tissues by directly acting on the pro-angiogenic signalling cascade in vascular endothelium (**Figure 6**).

It is well established that eNOS activation is beneficial for various types of vascular diseases such as PAD (Murohara et al., 1998; Luque Contreras et al., 2006). CTRP9 stimulates NO production and eNOS phosphorylation in various cells such as endothelial and epithelial cells (Zheng et al., 2011; Li et al., 2016). The vasorelaxant effects of CTRP9 are mediated through its ability to activate eNOS-dependent signaling pathway (Zheng et al., 2011). Consistent with these findings, this study showed that CTRP9 increased eNOS phosphorylation in cultured endothelial cells. Our data also showed that NOS inhibition reversed the actions of CTRP9 on endothelial network formation and migration. Furthermore, the stimulatory effects of CTRP9 on blood flow in ischemic muscle were abolished in eNOS-KO mice. These observations suggest that eNOS acts as a key mediator of the proangiogenic actions of CTRP9. Taken together, it is conceivable that eNOS is essential for the vascular protective actions of CTRP9.

Several reports demonstrated that CTRP9 activates AMPK and Akt signaling pathway in various cells such as endothelial cells (Peterson et al., 2013; Sun et al., 2017; Zheng et al., 2011; Kambara et al., 2015). CTRP9 promotes AMPK signaling pathway in skeletal muscle, leading to improvement of metabolic dysfunction (Peterson et al., 2013). CTRP9 also reduces endothelial injury in response to oxidized low-density lipoprotein through AMPK activation (Sun et al., 2017). It has been reported that CTRP9 induces endothelium-dependent vasorelaxation through the AMPK/Akt/eNOS signaling pathway (Zheng et al., 2011). We have reported that treatment of cardiac myocytes with CTRP9 suppresses hypoxia/reoxygenation-induced apoptosis and LPS-induced expression of proinflammatory cytokines through activation of AMPK (Kambara et al., 2015). Our *in vitro* study revealed that CTRP9 stimulated eNOS phosphorylation in endothelial cells through AMPK or Akt activation. In addition, CTRP9 deficiency contributed to reduced phosphorylation of AMPK, Akt, and eNOS ischemic muscle. Collectively, these data suggested that AMPK or Akt signaling pathway can be crucial for cardiometabolic protection by CTRP9.

In recent years, therapeutic angiogenesis has been developed as a novel strategy for patients with PAD (Raval and Losordo, 2013). Adipose-derived regenerative cells (ADRCs) are known to have differentiation ability into a variety of cell lineages, secrete many cytokines, and exert an angiogenic effect by implantation (Kondo et al., 2009a). ADRC implantation has been performed for CLI in several institutions, and favorable results have been reported (Lee et al., 2012; Bura et al., 2014). A recent study demonstrated that CTRP9 promotes proliferation, survival and migration of ADRCs, resulting in enhancement of cardioprotective effect of ADRC implantation (Yan et al., 2017). These findings suggest that ADRC implantation in combination with CTRP9 may be a highly effective strategy for therapeutic angiogenesis in PAD patients.

In conclusion, we found that CTRP9 functions as a novel proangiogenic factor. Thus, therapeutic approaches aimed at increasing CTRP9 production could be potentially useful for treating diseases associated with vascular insufficiency such as PAD.

## Data Availability Statement

All datasets presented in this study are included in the article/supplementary material.

## Ethics Statement

The animal study was reviewed and approved by The Institutional Animal Care and Use Committee in Nagoya University.

## Author Contributions

SY, RS, NOu, MH, TMa, HK, and TMu designed and carried out the studies. SY, RS, KO, TMa, and NOt analyzed the data. RS and NOu wrote the paper. All authors contributed to the article and approved the submitted version.

## Funding

This work was supported by Grant-in-Aid for Scientific Research A, and grants from Takeda Science Foundation to NOu. RS was supported with Grant-in-Aid for Scientific Research C.

## Conflict of Interest

The authors declare that the research was conducted in the absence of any commercial or financial relationships that could be construed as a potential conflict of interest.

## Abbreviations

*Ad*, adenovirus; *AMPK*, AMP-activated kinase; *β-gal*, β-galactosidase; *CTRP*, C1q/TNF-related protein; *eNOS*, endothelial nitric oxide synthase; *HUVECs*, human umbilical vein endothelial cells; *LDBF*, laser Doppler blood flow; *WT*, wild-type.
